# Poor sleep efficiency and daytime napping are risk factors of depersonalization disorder in female university students

**DOI:** 10.1016/j.nbscr.2020.100059

**Published:** 2020-10-14

**Authors:** Teresa Arora, Eman Alhelali, Ian Grey

**Affiliations:** a Zayed University, Abu Dhabi, United Arab Emirates; b Lebanese American University, Beirut, Lebanon

**Keywords:** Sleep quality, Daytime napping, Depersonalization disorder, University students, Sleep efficiency

## Abstract

**Objectives:**

Depersonalization is characterized by feelings of detachment from reality and has been associated with anxiety and depression, both of which have a bi-directional relationship with sleep. To date, few studies have directly examined the potential relationship between sleep and depersonalization, which was the primary objective of our study.

**Design/methods:**

A cross-sectional study of female, Emirati, university students (n = 100) was conducted. Participants completed the Pittsburgh Sleep Quality Index (PSQI), the Cambridge Depersonalization Scale (CDS) and the Hospital Anxiety and Depression Scale (HADS). Additionally, 36 of the 100 participants wore wrist actigraphy for two consecutive weekdays. Average sleep duration, and average sleep efficiency (SE; %) across the two nocturnal sleep episodes were calculated. Total number of sleep episodes were obtained from wrist actigraphy and sleep logs.

**Results:**

A significant, positive relationship was observed between PSQI global score and CDS total score (r = 0.21, p = 0.04). Actigraphy-estimated average nocturnal sleep duration was not significantly associated with the CDS. Compared to nocturnal sleepers only, those who undertook daytime naps had almost three times the risk of meeting the criteria for depersonalization disorder (OR = 2.95, 95% CI: 1.04–8.41), after adjustment. For each 1% increase in SE a 23% decreased risk of depersonalization was observed (OR = 0.77, 95% CI: 0.61–0.96), after adjustment.

**Conclusions:**

Sleep screening in young adults may help to ensure better detection and management of psychological health outcomes. Our findings need to be confirmed prospectively in larger samples and amongst different populations but reiterate the importance of sleep habits pertaining to mental health.

## Highlights

• We show a novel relationship between depersonalization and sleep in a non-clinical sample.
• Actigraphy determined poor sleep efficiency was significantly associated with subjective reports of depersonalization.
• Daytime nappers were ~3 times more likely to report depersonalization symptoms and meet the diagnositic criteria.

## 1 Introduction

Dissociation and its disorders have long been the focus of clinical and research interest (Janet, 1873/1973). The term dissociation is generally considered to refer to a broad spectrum of experiences on a continuum from mild detachment from immediate surroundings and the self to more severe detachment from physical and emotional experiences (Lynn et al., 2019). Central to the definition of dissociation is that key aspects of psychobiological functioning such as perception, memory, and identity which are normally seamlessly linked into consciousness are disturbed. Dissociative experiences interrupt the person's perception of continuity of normal psychological functioning and coincide with a fragmentation of the sense of coherence, unity and contiguity of one's self (Simeon, 2004). Such disruption takes one of two forms, typically classed as either positive or negative (Sierra, 2008). Positive dissociation symptoms relate to the appearance of aversive events into awareness such as flashbacks which intrude on the person's normal functioning. Conversely, negative symptoms disrupt normal conscious functioning via deficits in memory, sense of self and/or the ability to control different parts of the body (Spiegel et al., 2011).

Dissociation can be best understood as an umbrella term for a number of symptoms such as derealization, depersonalization, psychological numbing and disengagement, which vary along a continuum from non-pathological manifestations to more severe disturbances (van Heugten-van der Kloet et al., 2014). Derealization refers to the experience of feeling detached from ones immediate surroundings which may also feel unreal (Lynn et al., 2019). Depersonalization in contrast primarily refers to a sense of detachment from oneself and feelings of being an outside observer of the self. Though depersonalization and derealization are often considered as single most important symptoms in the spectrum of dissociative disorders, it features prominently in a host of other non-dissociative disorders including clinical depression, post-traumatic stress disorder, borderline personality disorder and conversion disorder (Lyssenko et al., 2018). However, dissociative symptoms have also been reported to occur in the absence of these disorders (Aderibigbe et al., 2001).

The etiology of dissociative phenomena remains a fiercely debated topic and, historically, trauma models have been the most widely proposed and accepted (Lynn et al., 2019). Central to the trauma model of dissociation, dissociation is viewed as an adaptive response following exposure to traumatic events and dissociation is postulated to serve a defensive function facilitating the person to avoid memories of aversive events (Gershuny and Thayer, 1999; Lynn et al., 2019). In a related variation of the trauma based model, dissociative experiences are conceptualized as an inhibitory response to help preserve adaptive behavior by attenuating anxiety and psychobiological hyperarousal under threat conditions (Sierra, 2008). In this model, both depersonalization and derealization may represent a generalization of responses to situations outside of immediate threat (Spiegel et al., 2011).

Indeed while a number of studies report a correlational relationship between the experience of trauma and subsequent symptoms of dissociation, the range of variations of correlations is reported as being highly variable (Briere, 2006; Lynn et al., 2019). Furthermore, other research indicates no history of trauma in sizeable numbers of individuals with dissociative disorders (Vogel et al., 2009). As such, there is a need to reconcile the high prevalence of experiences such as depersonalization with the rate of reported traumatic experiences, which are often substantially lower (Aderibigbe et al., 2001; Lynn et al., 2019). Finally, depersonalization has been reported as occurring in the context of sleep deprivation, migraine, and temporal lobe epilepsy (Lambert et al., 2002).The foregoing discussion would suggest that additional etiological factors for symptoms of dissociation may be in operation. One alternative proposal that has attracted increased attention, is the possible role of sleep, and in particular sleep disturbances, in relation to symptoms of dissociation (van Heugten-van der Kloet et al., 2015).

In respect of the association between sleep and dissociation, Watson (Watson, 2001, 2003) was amongst the first to postulate that disruptions to sleep may actually serve to intensify dissociative symptoms. Subsequent research indicating that acute dissociation symptoms intensify in response to sleep deprivation have lent support to this initial proposition. (Giesbrecht et al., 2007). These early findings have provided an impetus for more rigorous studies to be conducted, many of which lend increasing support to a possible causal relationship between disturbances in sleep and the occurrence and/or exacerbation of dissociative symptoms. For example, additional research has demonstrated that sleepiness may precede an increase in dissociative symptoms in young people and that such increases are not mediated by a deterioration in mood (van der Kloet et al., 2012). In addition, Poerio et al. (2016) examined the respective roles of sleep duration and sleep quality and the experience of dissociation. Their results indicate that sleep quality but not duration was a negative predictor of daily dissociation. More recently, one large scale study utilizing a non-clinical sample reported an association between experiences of depersonalization and sleep quality with poorer sleep quality being related to greater symptoms of depersonalization (Denis and Poerio, 2017). It also appears that improvements in sleep are associated with a reduction in dissociative experiences thereby lending additional support to a potential causal role of sleep disruption. Indeed, it has been shown that normalization of the sleep-wake cycle over a six-week period amongst inpatients with depression, anxiety and addiction resulted in decreased self-reported dissociative symptoms (van der Kloet et al., 2012). However, one general criticism of the above research is that focused primarily on correlational methodologies, which are inherently limited. One notable exception pertains to the work of van Heugten and colleagues. Using an experimental format, they subjected one group of randomly assigned healthy volunteers to 36 h of sleep deprivation and compared their performance on memory tasks for emotional material and self-reported symptoms of dissociation, to a matched control group (van Heugten-van der Kloet et al., 2015). They reported that sleep deprivation was associated with an increase in dissociative symptoms and that sleep loss also undermined memory of emotional material, particularly in highly dissociative individuals.

The question as to why disturbances in sleep appear to lend themselves to dissociative experiences has prompted researchers in the area to put forward a possible explanatory framework. Central to this account is that deficiencies in sleep parameters such as duration and quality are likely to result in a loosening of cognitive control which render the individual susceptible to “an influx of imaginative, dreamlike mentation in daily life that contributes to dissociative symptoms such as depersonalization and derealization” (van der Kloet et al., 2012b, p. 167). As such, a dysfunctional sleep-wake cycle may serve to promote episodes of dissociation by facilitating sleep-like mentation to permeate into waking consciousness, which “consequently fuels fantasy-proneness, and is associated with cognitive failures, and feelings of depersonalization/derealization” (Poerio et al., 2016).

The factors contributing to dysfunctional or labile sleep-cycles have themselves been the focus of a considerable body of research. While the importance of sleep on several indices of psychobiological functioning is rapidly increasing, the role of cultural practices and their respective impact on sleep is less well studied and understood. Anecdotal evidence from Middle East countries points to widespread variations in sleep-wake timings, which our groups data also confirms (currently under review). However, there remains a general paucity of empirical evidence surrounding sleep practices in this region. There is growing interest in the relationship between sleep, obesity and its comorbidities, as these chronic health problems disproportionally affect Middle East countries. Of the few studies that are available, the existence of poor sleep appears to be widespread in this region of the world. For instance, a Lebanese group recently observed that over 44% of a large cohort of adults reported the presence of symptoms of insomnia and that 34% reported the presence of insomnia (Chami et al., 2019). Other work points to a high prevalence of reduced sleep amongst Saudi adults, with approximately one in three adults reporting a sleep duration of less than 7 h per night (Ahmed et al., 2017). Other research points to a high rate of daytime sleepiness within this population (22% for females and 20% for males), which point to possible disruptions in the sleep-wake cycle for these respondents (Fatani et al., 2015). One further study of Saudi adolescents also indicated the presence of disrupted sleep, poor sleep quality and reversed weekend sleep cycles (Merdad et al., 2014). Some attempts have been made to identify the etiological factors implicated in sleep quality and in particular the role of poor sleep hygiene practices. For example, a high prevalence of behaviors associated with sleep disturbance such as lying down for a nap after lunch (88%) and sleeping in the car (74%) amongst Saudi medical students has been recently highlighted (Alodhayani et al., 2017). However, precise information regarding sleep timing and sleep variability across the diurnal cycle was not assessed in this study.

In the United Arab Emirates (UAE), a country culturally similar to Saudi Arabia, disruptions to sleep have also been documented (Mussa et al., 2019). Furthermore, another study showed that 34% of individuals recruited to a study were classified as poor sleepers based on the Pittsburg Sleep Quality Index(Bani-Issa et al., 2018). In particular, females showed a higher prevalence of poor sleep (62%) compared to males (38%). Such studies are limited by the self-report nature of the research and, what is notably absent is the use of more objective and precise sleep estimates, such as wrist actigraphy.

Previous studies that have examined sleep disturbances and symptoms of dissociation are also limited given that the primary focus has been on sleep duration. This neglects the potential role played by sleep quality and daytime napping. A focus on the number of sleep episodes and its relationship within symptoms of dissociation remains currently absent in the research literature, which is unfortunate given the role daytime napping plays in relation to a range of psychobiological variables. For example, longer daytime naps have been associated with cardiovascular disease (Yan et al., 2019), and overall mortality (Zhong et al., 2015). Given these specific relationships between sleep episodes and such outcomes, it is plausible to assume that increased numbers of sleep episodes may be differentially related to more specific psychological variables, such as experiences of depersonalization.

The current research sought to explore the potential relationship between various sleep parameters and self-reported experiences of depersonalization amongst a sample of female university students (i.e. a non-clinical sample). We hypothesized that self-reported and objective sleep estimates would be significantly associated with self-reported depersonalization in this sample. Consistent with previous research in this area, our study used previously validated self-report measures but also included wrist actigraphy to obtain objective estimates of sleep efficiency and total number of sleep episodes.

## 2 Material and methods

The study was approved by Zayed University (ZU) Research Ethics Committee. Undergraduate students from ZU, Abu Dhabi campus, were approached for study participation during a series of brief classroom presentations which highlighted specific details of the study. Posters advertising the study were displayed across different locations within the university. Written informed consent was obtained from those interested and willing to take part. Student volunteers were not compensated in any way for study participation. A total of 100 participants were recruited to the study and given that the study was exploratory, no formal sample size calculation was conducted. Inclusion criteria for the study were females, registered ZU students, and aged between 18 and 30 years. Exclusion criteria for the study were those not fluent in English, vulnerable populations, and/or illiterate.

In addition to obtaining demographic information (age, education level, nationality), the following questionnaires were administered to participants who were asked to indicate their responses on paper copies after full explanation and instructions were given by the researcher:

1. Cambridge Depersonalization Scale (CDS). This questionnaire was originally developed in 2000 (Sierra and Berrios, 2000). It is a comprehensive instrument including 29 items addressing the complaints associated with depersonalization disorder (DPD). The CDS captures the frequency and duration of DPD over the previous six months and has been previously validated and demonstrated good reliability. According to accepted cut points a CDS total score of >70 indicates the presence of depersonalization disorder (DPD). A total CDS score of ≤70 indicates an absence of DPD (i.e. healthy). In the present study, the Cronbach's alpha coefficient was high (Cronbach α = 0.97).
2. The Pittsburgh Sleep Quality Index (PSQI) is a self-rated questionnaire which assesses overall (global) sleep quality (Buysse et al., 1989). The PSQI is one of the most widely utilized tools for sleep quality assessment. Nineteen items generate seven components: (1) self-reported sleep quality, (2) sleep latency, (3) sleep duration, (4) habitual sleep efficiency, (5) sleep disturbances, (6) use of sleeping medication, and (7) daytime dysfunction. A PSQI score of >5 distinguishes poor quality sleepers, from good quality sleepers (≤5).
3. The Hospital Anxiety & Depression Scale (HADS) is commonly used to determine levels of anxiety and depression as a screening instrument (Zigmond and Snaith, 1983). It includes 14 items in total which are equally divided to assess levels of depression and anxiety. Self-reported responses are based on the previous week at the time of data collection. HADS is well validated and has shown extensive reliability in clinical and non-clinical populations. A score of ≤7 indicates ‘normal’ levels of anxiety (i.e. healthy); a score of 8–11 implies a ‘borderline’ case; and ≥12 indicates an ‘abnormal’ case for both anxiety and depression. In the present study, the Cronbach's alpha coefficient was high for anxiety (Cronbach α = 0.88) and depression (Cronbach α = 0.83).

In order to objectively estimate sleep in a free-living environment, wrist actigraphy (GT3× + BT, Actigraph, Pensacola, FL, USA) was administered (non-dominant hand) to willing participants for two consecutive weekdays and nights with concurrent sleep logs. The sleep log asked participants to estimate bed, sleep, and wake times as well as document the times (to and from) for any daytime naps. The data were downloaded according to the manufacturer's software (ActiLife, version 6). Sleep scoring was conducted based on Cole-Kripke algorithms and sleep logs were used to modify sleep-wake timings and daytime naps. Sleep efficiency (%), and sleep duration (minutes) were obtained from the actigraph. Number of sleep episodes per 24-h period was obtained from wrist actigraphy in combination with the sleep logs. Sleep episodes included nocturnal and daytime napping which were totaled across the two-day actigraphy period. Average nocturnal sleep duration and sleep efficiency was calculated by summing the nocturnal sleep duration and efficiency, respectively, and dividing by two.

### 2.1 Statistical analysis

All statistical analyses were performed using Stata SE, version 13. For continuous variables, distribution of the data checked for normality. Non-parametric tests were conducted for non-normally distributed data. Parametric tests were conducted for normally distributed data. Descriptive statistics were obtained including mean and standard deviation for normally distributed data. Median and interquartile range (IQR) were reported for non-normally distributed data. A series of bivariate correlations were performed using Spearman's Rho. Bivariate correlations were used to assess the relationships between self-reported sleep quality (global PSQI score), psychological health (anxiety and depression), and depersonalization. Chi squared analysis was performed to assess the relationship between self-reported sleep quality (PSQI score of >5 versus ≤5) and depersonalization (CDS total score of >70 versus ≤70). An independent *t*-test was conducted to assess the mean sleep duration (according to self-report question on PSQI) according to those with and without depersonalization disorder (based on the CDS established cut point). Finally, a series of logistic regression analyses were performed to assess if average nocturnal sleep duration and sleep efficiency (%) as well as the number of sleep episodes (derived from wrist actigraphy) was associated with risk of depersonalization disorder (using the recognized CDS cut points). Univariate models were initially conducted and subsequent multivariate analyses were performed to adjust for potential confounding factors including PSQI (dichotomized), level of anxiety and level of depression, based on previous empirical evidence. As the sample was homogeneous in terms of age, nationality and education level these were not adjusted for in the regression analysis. Moreover, gender was not adjusted for as the sample was 100% female. In order to assess the possibility of self-selection bias, we also examined potential differences for subjective sleep quality (PSQI global score), total CDS score, HAD depression and anxiety scores between those who wore wrist actigraphy and those who did not.

## 3 Results

The sample characteristics are presented in Table 1 and show that the majority of the participants were aged between 18 and 25 years (98%) and were from the UAE (99%). Additionally, the majority of the participants scored ‘abnormal’ for anxiety (43%), although 59% were considered ‘normal’ for depression, according to the HADS. The mean sleep duration was 6.1 ± 1.9 h, and the average PSQI global score was 8 ± 4. Most of the participants had poor sleep (75%) and the median for the total CDS score was 58 (IQR: 26–87).

**Table 1:** **Sample characteristics of 100 female university students**.

Characteristic	
Age (years)	
18–25	98%
26–30	2%
**Gender:**	
Female	100%
**Nationality**	
United Arab Emirates	99%
Other	1%
**Education Level**	
High School	2%
Undergraduate	95%
Post-graduate	3%
**HADSA**	
Normal (0–7)	22%
Borderline (8–10)	35%
Abnormal (11–21)	43%
**HADSD**	
Normal (0–7)	59%
Borderline (8–10)	32%
Abnormal (11–21)	9%
**PSQI**	
Sleep Duration	6.1 ± 1.9
PSQI Global	8 ± 4
**PSQI (binary)**	
Good sleep quality	25%
Poor sleep quality	75%
**CDS**	
CDS Total	58 (26–87)
CDS Duration	30 (16–53)
CDS Frequency	25 (12–37)
**Actigraphic sleep (n = 36)**	
Average nocturnal sleep duration (minutes)	452 ± 100
Average nocturnal SE	90 (87–93)
Number of sleep episodes over two-day period	2 (2–3)

Data are presented as percentage, mean (standard deviation), or median (Interquartile range).

HADSA= Hospital Anxiety & Depression Scale (Anxiety); HADSD= Hospital Anxiety & Depression Scale (Depression); PSQI= Pittsburgh Sleep Quality Index; CDS= Cambridge Depersonalization Scale; SE = sleep efficiency.

Table 2 depicts the bivariate correlation analyses between global PSQI score and psychological health as well as depersonalization.

**Table 2:** Bivariate correlations between global PSQI score, anxiety and depression, and depersonalization in 100 female university students.

	Global PSQI	HADS Anxiety	HADS Depression	CDS frequency	CDS duration	CDS total
**Global PSQI**	1.00	0.233^a^	0.134	0.153	0.190	0.212^a^
**HADS Anxiety**	0.233^a^	1.00	0.445^c^	0.258^b^	0.343^c^	0.355^c^
**HADS Depression**	0.134	0.445^c^	1.00	0.328^c^	0.298^b^	0.346^c^
**CDS frequency**	0.153	0.258^b^	0.328^c^	1.00	0.718^c^	0.802^c^
**CDS duration**	0.190	0.343^c^	0.298^b^	0.718^c^	1.00	0.963^c^
**CDS total**	0.212^a^	0.355^c^	0.346^c^	0.802^c^	0.963^c^	1.00

a p < 0.05.

b p < 0.01.

c p < 0.001.

CDS= Cambridge Depersonalization Scale. PSQI= Pittsburgh Sleep Quality Index. HADS=Hospital Anxiety and Depression Scale.

Chi square analysis revealed a borderline significant association between sleep quality and depersonalization, where χ^2^ = 3.70, p = 0.05. Of the total sample, 86.11% of those that met the criteria for depersonalization disorder had poor quality sleep. The majority (80%) of those with good quality sleep did not meet the criteria for depersonalization disorder. The minority (13.89%) of those with depersonalization disorder had good quality sleep, according to established PSQI cut points.

An independent *t*-test showed no significant mean difference in self-reported nocturnal sleep duration according to depersonalization disorder status, where t (98) = 0.46, p = 0.65. Average self-reported sleep duration for those without depersonalization disorder was 6.21 ± 1.95 h (n = 64) compared to those with depersonalization disorder (6.03 ± 1.84 h; n = 36).

Table 3 shows the results from a series of univariate and multivariate logistic regression analyses used to predict if various sleep parameters (derived from wrist actigraphy) were predictive of a significant risk for depersonalization disorder. The findings show that average nocturnal sleep duration across the two days/nights the actigraphy was worn was not significantly associated with the presence of depersonalization disorder, either before (OR = 1.00 [95% CI: 1.00–1.01], p = 0.30), or after adjustment for subjective sleep quality, level of anxiety and depression, where OR = 1.00 (95% CI: 0.99–1.01, p = 0.78). Conversely, objective estimates of sleep efficiency (%) was significantly associated with depersonalization disorder after adjustment, where OR = 0.77 (95% CI: 0.61–0.96), p = 0.02. The greatest effect was observed for increasing frequency of sleep episodes across the two-day actigraphy period. After adjustment, each additional sleep episode was associated with an almost three-fold increased risk of the presence of depersonalization disorder, OR = 2.95 (95% CI: 1.04–8.41), p = 0.04.

**Table 3:** Univariate and multivariate logistic regression analyses to examine the associations between objective sleep estimates and depersonalization disorder.

	Model 1	Model 2
Average nocturnal sleep time (minutes)	1.00 (1.00–1.01)	1.00 (0.99–1.01)
Average nocturnal sleep efficiency (%)	0.81 (0.68–0.98)^a^	0.77 (0.61–0.96)^a^
Number of sleep episodes	3.20 (1.29–7.93)^a^	2.95 (1.04–8.41)^a^

Data are presented as odds ratio (OR) and 95% confidence intervals (CI).

Model 1 = unadjusted/univariate.

Model 2 = adjusted for PSQI, anxiety, and depression.

a p < 0.05; **p < 0.01; ***p < 0.001.

There was no significant difference between participants who wore wrist actigraphy compared to those who did not for the global PSQI score (t (98) = -0.77, p = 0.44), the total CDS score (t (98) = 0.21, p = 0.84), HADS depression score (t (98) = -1.53, p = 0.13), or HADS anxiety (t (98) = 0.22, p = 0.83).

All sleep estimates were based on the two-day/night wrist actigraphy period.

## 4 Discussion

Etiological models of disorders are critical to guide appropriate intervention. The idea that disturbances in sleep are associated with symptoms of dissociation has been gaining increasing traction owing to the growing body of empirical support in this domain. To date, at least one large scale study has provided evidence for this association in a non-clinical sample (Denis and Poerio, 2017) and although using different measures, results of the current study lend additional support to these earlier findings.

Perhaps the most pertinent finding in the current study concerns the relationship between sleep quality and depersonalization. Whilst recent research points to a potential role played by sleep deprivation and subjective experiences of depersonalization (Poerio et al., 2016), closer investigation of the role between key indicators of sleep quality, such as sleep efficiency and frequency of sleep episodes, remains sparse in literature to date. However, consistent with recent research (Denis and Poerio, 2017) sleep duration was not found to be significantly associated with depersonalization, as assessed by the CDS though global scores on the PSQI and the CDS were correlated, albeit weakly. Specifically, there was no significant difference in average nocturnal sleep duration between respondents relative to depersonalization status. When respondents were divided into high and low scores on depersonalization, both groups averaged similar sleep duration. However, sleep efficiency, and increasing frequency of sleep episodes, were found to be associated with increased depersonalization, even across a short two-day period when wrist actigraphy was deployed. This adds to the existing body of research in a number of important ways. Firstly, it parallels previous research regarding the relationship between sleep efficiency and depersonalization. It appears that not only is sleep efficiency linked to experiences of depersonalization, but that as the number of sleep episodes increase, so do self-reported symptoms of depersonalization. The bulk of previous research has primarily focused on the role played by sleep deprivation in relation to depersonalization and, though the respective number of studies examining this relationship remains small, they do suggest that sleep deprivation is linked to depersonalization. It would appear, based on our results, that poor sleep efficiency and increasing frequency of sleep episodes, are also linked. Some researchers in the field have proposed that sleep quality, rather that sleep duration, acts to ‘potentiate’ dissociative experiences (Poerio et al., 2016). This approach is given credence in the broader context of differing topographies of sleep disturbance and dissociative experiences. Specifically, it appears that anomalous sleep experiences (e.g., excessive REM sleep, sleep paralysis, narcoleptic symptoms, daytime micro-sleeps, and hypnagogic and hypnopompic hallucinations) also render the individual more susceptible to dissociative experiences (van der Kloet., 2012). The proposed commonality across these sleep disturbances is that each results in difficulty regulating and controlling conscious states leading to cognitive disinhibition. Such disinhibition is postulated as having a biological basis in the form of a failure of pre-frontal disinhibition (Lynn et al., 2019). Collectively, this corpus of research points to the possibility of a non-trauma pathway to the onset and exacerbation of dissociative experiences.

The potential role of sleep efficiency, and in particular the types of sleep disturbance identified earlier, in facilitating depersonalization should not come as a surprise given the emerging links between alternations in sleep-wake cycles such as daytime napping and a range of physiological conditions. A number of studies have now demonstrated that daytime napping is associated with a number of potentially adverse health effects such as increased mortality (Dautovich et al., 2012), decreased renal functioning (Ye et al., 2019), increased risk of Parkinson's Disease (Leng et al., 2018), type 2 diabetes mellitus (Lam et al., 2010), and cardiometabolic risk (Buxton et al., 2018). Whilst the majority of research to date has focused on physiological conditions, some research has examined the role of napping in the context of specific psychological conditions, though not depersonalization. For example, the presence of depressive and anxiety disorders appears to be related to an increased likelihood of napping during the day (Leger et al., 2019). It is feasible to suggest that daytime napping may be driven by short or disrupted nocturnal sleep. However, our study observed a relationship between anxiety and sleep duration but not depression.

Owing to established links between affective disorders and sleep disturbance, the current study also investigated the relationship between various parameters of sleep and indices of depression, anxiety and depersonalization specifically. The prevalence or incidence of depression has been previously associated with increased sleep disturbances (Riemann et al., 2001). For example, individuals with depression exhibit alterations in sleep architecture (reduced slow wave sleep), as well as sleep efficiency. Conversely, insomnia patients experience elevated symptoms of depression compared to healthy individuals. However, in the current study no significant relationship was observed between depression and global PSQI score. This suggests that the high rate of poor sleep quality is unlikely to be attributable to the presence of depressive symptomology in our sample. In contrast, a significant relationship was observed between anxiety and global PSQI. Traditionally, sleep disturbance has been frequently viewed as a consequence of anxiety. However, sleep disturbances may not merely be a symptom, but also an etiological factor in the development of anxiety (Zenses et al., 2019). For example, prospective studies demonstrate that sleep disturbances at baseline increase the likelihood of developing subsequent symptoms of anxiety (Breslau et al., 1996; Jansson-Frojmark and Lindblom, 2008). More recent experimental work suggests that experimentally induced sleep deprivation, for as little as one-night, serves to increase subjective threat expectancies, which is a recognized cognitive bias for the onset and maintenance of anxiety (Zenses et al., 2019). As such, it is possible that participants in our study with poor sleep quality may have increased these subjective threat experiences resulting in higher rates of self-reported anxiety. This notion, of course, requires further investigation. However, while sleep deprivation *per se* appears to be linked to anxiety, what is less clear is the role of diminished sleep quality and its relationship with anxiety. It is possible that chronic poor sleep quality mimics the effects of acute sleep deprivation for some individuals in respect of anxiety, but this issue remains current unanswered.

Consistent with previous research, we found that, similar to other countries in the Middle East such as Saudi Arabia and Lebanon, a large proportion of respondents reported high levels of poor sleep quality. Methodological differences should, however, be noted surrounding the assessment of sleep quality in previous studies. Nonetheless, empirical evidence suggests that poor sleep quality, amongst adolescents and adults in Middle East countries, is widespread (Bani-Issa et al., 2018; Chami et al., 2019). Though the current study was restricted to female participants only, previous studies conducted in the Middle East suggest that females in this region, tend to report poorer sleep quality. For instance, one group has demonstrated that females in Saudi Arabia had a higher prevalence of poor sleep compared to males (Bani-Issa et al., 2018). In the current study, rates of poor sleep were in excess of those reported in Saudi Arabia (75% and 62% respectively), despite using the same instrument (PSQI). This raises legitimate questions in relation to the etiological factors behind such a high prevalence of poor sleep. Research emerging from the region to date remains largely limited to investigations of co-morbidities with physiological abnormalities in domains such as endocrine functioning and cardiovascular conditions. It is reasonable to assume, however, that a host of cultural and socio-demographic factors also play a role, given that substantial variations in subjective sleep quality exist worldwide (Knutson, 2013). Future work in this region of the world would undoubtedly benefit from a closer examination of cultural variables and their relationship with various indices of sleep quality.

Whilst the current study lends support to an association between sleep and depersonalization using a non-clinical sample, there are some study limitations that we acknowledge. First, although wrist actigraphy was administered to estimate sleep in a free-living environment, the uptake of participants agreeing to this was limited. Of the total sample, 64% initially agreed to wear the actigraph, but only 36 (56%) attended to the study visit to receive the equipment. This is an important point which could indicate self-selection bias whereby those with poorer sleep may have been more likely to participate. However, in our analysis, we found no evidence of a significant difference between the two groups for subjective sleep quality, depersonalization, anxiety or depression. Furthermore, whilst the device was administered to capture weekdays, participants were only asked to wear it for two consecutive days/nights to ensure the study was not overly burdensome. Wrist actigraphy has been previously recommended for two days/nights and five days/nights for estimating sleep percent and sleep efficiency, respectively (Aili et al., 2017). Whilst our study captured two nights of actigraphy data, this objective sleep estimate method is superior to subjective reports alone. We did not ask participants to indicate if their sleep habits were similar or dissimilar to their usual habits whilst wearing the wrist actigraphy. However, previous research indicates that there is no first night effect of wrist actigraphy (Arora et al., 2016). Second, the sample was limited to female university students, thus the findings may not be generalizable to other populations. Future studies should investigate the potential relationships in other groups, including males and older adults where sleep quality is more likely to be impaired. Third, the study was cross-sectional, therefore no causal explanation can be determined. Additional studies are needed in this area to identify if poor quality sleep and frequency of sleep episodes cause or contribute to the onset and/or progression of depersonalization disorder. In addition, the questionnaires administered were in English but the national language is Arabic. However, as the university curriculum is taught in English, this should not have posed language barrier concerns, although we acknowledge that the meaning of some questions may have been misinterpreted by participants. Researchers conducting studies in this area in the future would benefit from appropriately translated research measures to reduce this potential source of error.

## 5 Conclusions

Self-reported and objective indicators of sleep quality are significantly associated with depersonalization. In contrast, sleep duration was not significantly associated with dissociative symptoms. The study findings also supported previously published data in the area of sleep quality and mental health suggesting that sleep is directly related to anxiety. Future recommendations include repeating the study to include other groups to ensure generalizability across different populations/cultures. Wrist actigraphy should be administered for at least one-week and a longitudinal study could be conducted to establish causality. Should these studies reveal that sleep impairment promotes the onset and/or exacerbation of depersonalization disorder then this is likely to have implications for the treatment and management of patients in mental health settings. Moreover, based on our findings, we recommend that universities incorporate sleep assessment for students as part of in-house health and wellness programs.

## Funding sources

This research did not receive any specific grant from funding agencies in the public, commercial, or not-for-profit sectors.

## CRediT authorship contribution statement

**Teresa Arora:** Conceptualization, Methodology, Validation, Formal analysis, Investigation, Resources, Data curation, Writing - original draft, Writing - review & editing, Visualization, Supervision, Project administration. **Eman Alhelali:** Conceptualization, Methodology, Validation, Formal analysis, Investigation, Data curation, Writing - original draft. **Ian Grey:** Conceptualization, Methodology, Validation, Writing - original draft, Writing - review & editing.

## Declarations of competing interest

None.
